# Oligogenic Cardiomyopathy

**DOI:** 10.20517/jca.2021.27

**Published:** 2022-01-01

**Authors:** Ali J. Marian

**Affiliations:** Center for Cardiovascular Genetics, Institute of Molecular Medicine and Department of Medicine, University of Texas Health Sciences Center at Houston, Houston, TX 77030, USA.

We are grateful to Dr. McKenna and colleagues for discussing our recently published article in *The Journal of Cardiovascular Aging* in their editorial article^[1,2]^. We would like to clarify a few points:

Dr. McKenna and colleagues interpret the histology figure to diagnose cor adiposum in family member III-4 who died suddenly and was found to have extensive fibro-adiposis of the right ventricle^[1]^. The authors apparently made their diagnosis indicating that fibrosis is necessary for the diagnosis of arrhythmogenic right ventricular cardiomyopathy (ARVC). We had described the histological data as “fibro-fatty infiltration of the right ventricle, encompassing 50% to 80% of the right ventricular wall thickness”^[1]^. However, we had not included specific staining for myocardial fibrosis. We provide Masson trichrome-stained myocardial sections, which show unequivocal evidence of myocardial fibrosis along with the excess adipocytes [Figure 1]. We also note that individual III-4 had pathogenic and likely pathogenic variants (PVs/LPVs) in the *PKP2* and *DSP* genes, which are well-established causes of ARVC. Furthermore, cor adiposum does not exclusively and extensively involve the right ventricle, as observed in individual III-4, without involving the left ventricle. Thus, the data firmly refutes the diagnosis of cor adiposum and confirms the diagnosis of the classic ARVC.

Dr. McKenna and colleagues imply that the PVs/LPVs, except the canonical splice site variant c.39893-1G>A in the *TTN* gene, were unlikely modifiers of the phenotype. Apparently, this impression is based on the presence of these variants in the general population. The argument is based on a deterministic approach in analyzing the clinical significance of the genetic variants. Over the last decade, we have advocated that the probabilistic approach should be used in interpreting the phenotypic impact of the genetic variants^[3,4]^. Genetic variants exert a gradient of effect sizes that range from large to clinically indiscernible. To expand, the effect size inversely correlates with the population frequency of the variant. Rare variants, defined as the variants with a minor allele frequency (MAF) of < 0.01 in the general population, typically have large effect sizes, whereas the common variants (MAF > 0.01) exert small effect sizes. The causal variant is the rarer than the population frequency of the disease and has the largest effect size. Hence, the causal variant co-segregates with the phenotype, albeit with a variable and age-dependent penetrance. The modifier variants, when heterozygous, exert smaller effect sizes than the causal variants and are neither necessary nor sufficient to cause the disease, but whenever present, they influence the phenotypic expression of the disease. Consequently, the modifier variants are expected to be also detected in the general populations and not cause the disease of interest in the absence of the causal variant.

Dr. McKenna and colleagues state that PVs/LPVs in the *SCN10A* and *AKAP9* gene, reported in the family, were unlikely to contribute to the phenotype because they are associated with ion channel disease but not cardiomyopathy. The authors’ notion is likely a simple oversight of the fact that arrhythmias are major phenotypic features of cardiomyopathies. Consequently, PVs/LPVs in genes encoding ion channel genes could serve as susceptibility alleles to arrhythmias in patients with cardiomyopathies (reviewed in^[5]^).

To conclude, an unambiguous ascertainment of causality or the modifier effect(s) of PVs/LPVs in a member or members of a family is almost impossible. However, based on the probabilistic approach to the assessment of the clinical significance of the PVs/LPVs, the *PKP2* and *DSP* variants are most likely the causal variants for ARVC in individual III-4. Likewise, the PVs/LPVs in other genes involved in cardiomyopathies and arrhythmias were likely modifiers of the phenotype, including susceptibility to cardiac arrhythmias. Thus, our findings are in accord with the fact that the phenotype in genetic diseases, including those with Mendelian patterns of inheritance, is the cumulative effects of non-linear and stochastic interactions among multiple PVs/LPVs in genes pertinent to various aspects of the disease and the environmental (non-genetic) factors.

## Figures and Tables

**Figure 1.**
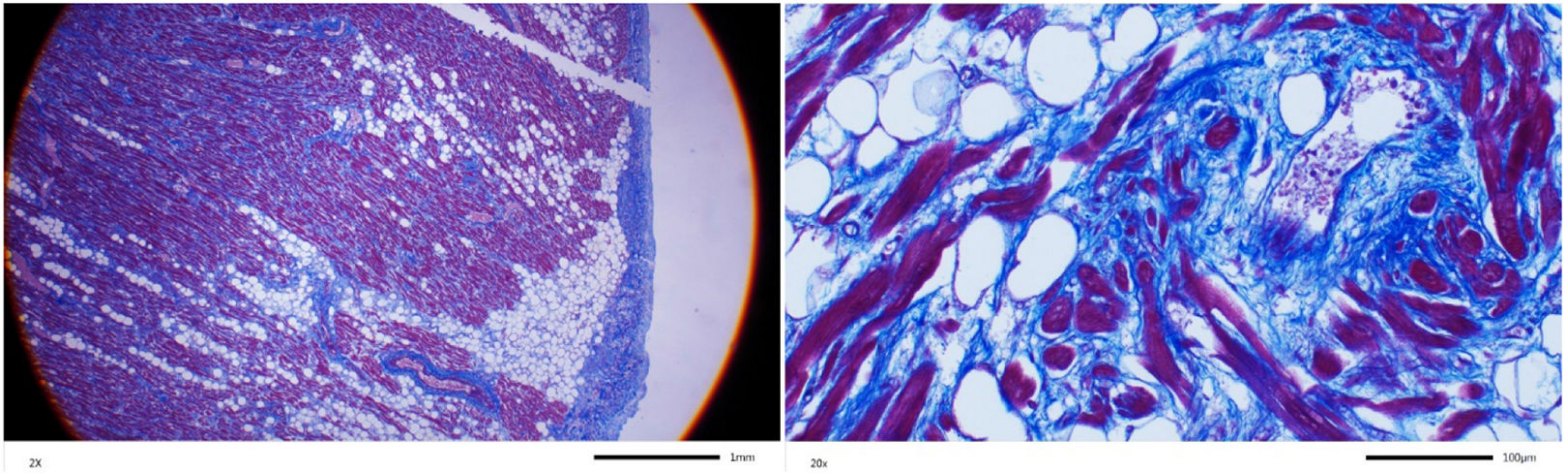
Masson trichrome-stained thin myocardial sections obtained from the heart of individual III-2 at autopsy. Low and high magnification panels are shown. Red color identifies myocytes, blue color represents fibrosis and white circles adipocytes.
